# The prevalence of non-communicable diseases and related risk factors in young adults in the Caribbean islands: a scoping review

**DOI:** 10.1186/s12889-026-27072-2

**Published:** 2026-03-28

**Authors:** Matthew J. Savage, Natalie Darko, Philip J. Hennis, Ruth M. James, Neval Grazette, Trevor S. Ferguson, Shelly McFarlane, Heather Armstrong, Horace Cox, Ian Varley

**Affiliations:** 1 SHAPE Research Group, School of Science and Technology, Nottingham Trent University, Nottingham, United Kingdom; 2 Diabetes Research Centre, University of Leicester, Leicester General Hospital, Leicester, United Kingdom; 3 Caribbean Institute for Health Research, The University of the West Indies, Kingston, Jamaica; 4 The Caribbean Public Health Agency, 6-18 Jamaica Blvd, Federation Park, Port of Spain, Trinidad & Tobago; 5 NIHR Leicester Biomedical Research Centre, Leicester, United Kingdom

**Keywords:** Health, NCDs, Obesity, Diet

## Abstract

**Introduction:**

Non-communicable diseases (NCDs) contribute to 70% of deaths across the Caribbean. This is driven by an aging population, increased urbanisation and a high susceptibility to climate change and natural disasters. Young adults represent a significant proportion of the Caribbean population and are expected to be disproportionately affected by the burden of NCDs in the near future. However, data surrounding NCD risk and prevalence in young adults across the Caribbean is limited. This scoping review aims to examine the current evidence on NCDs and their associated risk factors within this population.

**Methods:**

The following databases were searched from inception until June 2024: PubMed, Scopus, and Web of Science. Studies with data related to NCD prevalence, physical risk factors for NCDs, and behavioural risk factors for NCDs in people aged 17–30 in countries within the Caribbean islands were included. Data related to study design, population characteristics, outcome measures, and key findings were extracted.

**Results:**

11 studies were included in the final review. The majority were conducted in Jamaica (*n* = 5), with Trinidad and Tobago, Haiti, Grenada, Cuba, and Puerto Rico also represented. Only the prevalence of T2DM (0.0-2.9%), chronic kidney disease (CKD) (8.3%) and cardiac disease (1.0%) was reported. The most common physical risk factor was hypertension (1.5–20.9%) and low [HDL] was the most prevalent risk factor (15.9%-47.0%). Few studies reporting behavioural risk factors indicated that poorer behaviours were associated with increased prevalence of physical risk factors. Gender differences were observed, women generally exhibited a greater prevalence of physical and behavioural risk factors.

**Conclusions:**

This scoping review highlights the need for further research surrounding NCD risk and prevalence among young adults across the Caribbean. Generating further data on this topic will aid in developing targeted health-based initiatives to reduce the current and future burden of NCDs across the region.

**Supplementary Information:**

The online version contains supplementary material available at 10.1186/s12889-026-27072-2.

## Introduction

Non-communicable diseases (NCDs) are conditions that are not directly transmitted between individuals and typically develop as a result of a combination of genetic, environmental, and behavioural factors [1, 2]. These diseases are the leading cause of morbidity and mortality worldwide, responsible for over 40 million deaths each year [1]. Among the most prevalent NCDs are cancers, cardiovascular diseases and type 2 diabetes mellitus (T2DM) which collectively account for the majority of NCD-related deaths [1, 3, 4]. The global burden of NCDs has significantly increased in recent times with the number of annual deaths related to NCDs increasing by 5 million between 2006 and 2016 [4], and it is expected that this will continue to rise in line with an ageing global population [5]. This is amplified further in countries with a poorer socioeconomic status, within which 77% of NCD-related deaths currently occur [1] and where the burden of NCDs will likely exceed 80% in the near future [6].

The Caribbean islands are comprised of twenty-six countries and territories located within the geographical location of the Greater and Lesser Antilles and bordering the Caribbean Sea. All of which have unique characteristics related to size, landscape, resources, and health monitoring capabilities [7]. In the Caribbean, NCDs represent the most significant challenge related to health and disease burden and are attributable to 70% of deaths across the region [8, 9]. This exceedingly high burden of NCDs may be, in part, due to high levels of urbanisation throughout the region with several countries classified as Small Island Developing States (SIDS) [10]. These territories are often defined by their modest size, constrained resources, and susceptibility to external economic and environmental disruptions [10]. Limited land availability in many of these nations has resulted in the development of informal settlements or urban areas marked by elevated unemployment and poverty rates [10]. These underserved communities frequently struggle with inadequate housing and a lack of essential infrastructure to promote health and well-being [10]. The social, cultural, and economic diversity across the region may also play a significant role in the development of NCDs, with many Caribbean nations experiencing political turbulence, accelerated economic development, and deep-rooted inequalities leading to significant challenges in accessing resources and services required to reduce the burden of NCDs [11].

The Caribbean islands are also uniquely susceptible to natural disasters and are highly impacted by climate change [12, 13]. In Latin America and the Caribbean, temperatures are expected to increase by 4.5 °C by 2071 compared to temperatures from 1951 to 1980 [14]. Furthermore, tropical glaciers are expected to reach complete deglaciation due to periods of increased warming, and projections suggest that sea levels will rise by ~ 0.2–1.1 mm across the region [14]. The environmental consequences of these changes include greater incidence of extreme heat (i.e., droughts), periods of excessive precipitation (i.e., flooding), and a higher occurrence of natural disasters (e.g., hurricanes, earthquakes, tsunamis, and tropical cyclones) [14]. Ultimately, this will negatively impact people living in the region through reduced biodiversity and increased challenges in accessing natural resources [15, 16], and reduced food and water safety [17] leading to an increased risk of developing respiratory disorders and contracting waterborne and vector-borne diseases [18]. Furthermore, there is growing concern that sustained periods of extreme weather events will lead to a decrease in tourism, reduced opportunity to invest in sustainable energy, and impaired ability to produce crops for export, providing further socioeconomic challenges to reducing NCD risk [18].

The chronic nature of NCDs mean that the development of such disorders is more likely during advanced stages of the lifespan [19]. Across the Caribbean, recent data describe an ageing population with the proportion of those aged ≤ 15 years declining and the proportion of those aged 25–64 years increasing [20] providing some explanation to the rise in NCD prevalence across the region [21]. However, while NCD diagnosis often occurs in mid-late adulthood, the behavioural and lifestyle habits related to the onset of such diseases are often established during early adulthood [22–28]. Importantly, the proportion of young adults has increased substantially in recent years with this demographic comprising ~ 20% of the general population [29]. However, due to increased urbanisation across the Caribbean, young adults face reduced access to resources that support healthy behaviours [30]. Additionally, young adults are often excluded from health-based initiatives due to limited funding, low interest, limited perceived relevance, and/or the burden of participation [31]. As such, there is currently a dearth of information surrounding NCD risk and prevalence in young adults residing in the Caribbean. Limited data suggests that at least 40% of this demographic engage in excessive alcohol use, 60% are physically inactive, and 16% smoke [32, 33]. However, there is yet to be a study that synthesises knowledge of existing data surrounding the prevalence of NCDs and related risk factors in this population.

Young adults are predicted to be disproportionately affected by the burdensome consequences of NCDs in the near future [34]. Understanding the current landscape of data available will identify knowledge gaps and inform stakeholders of those who may benefit from increased funding and resources to develop future health-based initiatives and programs that will reduce the burden of NCDs in Caribbean young adults. The aim of this scoping review was therefore to examine existing literature surrounding the prevalence of NCDs and related risk factors in young adults in the Caribbean.

## Methods

This scoping review was reported in accordance with the Preferred Reporting Items for Systematic Reviews and Meta-Analyses Scoping Reviews (PRISMA-ScR) guidelines, the checklist for which is available in Supplementary data 1. An initial protocol was created prior to conducting the study but was not registered.

### Inclusion criteria

Complete manuscripts of original published research that included primary data and were specific to the research question were eligible for inclusion in the review.

Participants were young adults from the Caribbean islands. These were classified by age as being 17–30 years old in line with other literature in the area investigating the prevalence of NCD risk in young adults [35]. Studies from the Caribbean islands included countries within the geographical location of the Greater and Lesser Antilles and bordering the Caribbean Sea. The search included studies investigating the prevalence of NCDs or associated physical risk factors in the population of interest. Where appropriate, behavioural risk factors were also included but only as secondary outcomes in addition to physical measures.

### Search strategy

Three data bases were searched on June 10th 2024 without restriction on language or dates. These were as follows:

- Scopus
- PubMed
- Web of Science

Further details surrounding the search strategy are available in Table 1.

**Table 1:** Data retrieved from each database.Data retrieved from each database

Database	Search date	Database year of publication range	Number of records retrieved	Number of records following removal of duplicates
Scopus	June 10th 2024	1970–2024	311	309
PubMed	June 10th 2024	1959–2024	53	30
Web of Science	June 10th 2024	1993–2024	53	27

### Search terms

Terms related to the population of interest (e.g., young adults; young people), NCDs (e.g., Non-communicable diseases; Asthma; Heart Disease; Stroke), health related behaviours (e.g., Health Behaviours; Health-Related Behaviour), and the Caribbean (e.g., Caribbean islands; Anguilla; Antigua and Barbuda; Aruba; The Bahamas; Barbados) were included in the search strategy. The full list of terms included in the search strategy is available in Supplementary data 2.

### Identifying sources of evidence

Covidence software was used to screen all articles and to extract data (Covidence systematic review software, Veritas Health Innovation, Melbourne, Australia. Available at www.covidence.org). The title and abstracts of relevant literature were screened by two authors independently. Communication surrounding inclusion of articles occurred where necessary to resolve conflicts. The full text of included articles was then screened by the same two authors independently. Again, the authors resolved disagreements through verbal communication to confirm eligibility and reach a final consensus.

### Data extraction and charting

Data were extracted into a standardised data extraction template in Microsoft Excel. Data were extracted to examine participant characteristics (age & sex) and the prevalence of NCDs and/or associated risk factors.

## Results

**Fig. 1:**
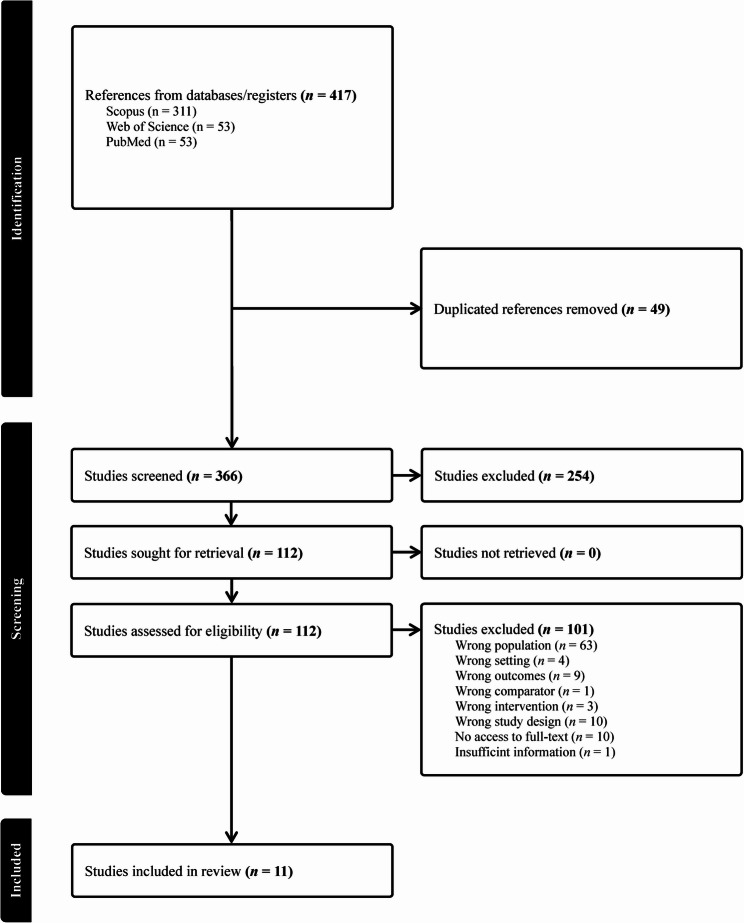
Flow diagram of study eligibility process

A total of 417 studies were identified through searches of electronic databases. Following the removal of 49 duplicates, 366 studies were screened using the title and abstract. Of these, 112 met the inclusion criteria. The full texts were then screened for eligibility and 101 were excluded for various reasons such as: not including data specifically in young adults, not including participants residing in the Caribbean, and insufficient information surrounding the prevalence of NCDs or physical risk factors. The reference lists of the full texts were also screened for additional articles. A total of 11 articles met the inclusion criteria and are included in data charting in this study. One study was originally written in Spanish and was translated into English [36]. Further information surrounding the eligibility process is provided in Fig. 1.

### Study characteristics

Information surrounding study characteristics is provided in Table 2. Studies analysed were published between the years 2010–2024. Five studies were conducted in Jamaica [33, 37–40], two in Trinidad and Tobago [41, 42], and the remainder in Haiti [43], Grenada [44], Cuba [36], and the USA and Puerto Rico [45]. Most studies employed a cross-sectional design (*n* = 9) [33, 36–39, 41–44] and two studies used longitudinal designs [40, 45]. Four studies utilised a cohort study design [37, 40, 43, 44] and two studies utilised retrospective data [41, 44]. Participants in all studies were aged between 17 and 30 years and most studies included a higher proportion of females compared to males [33, 38–42, 44, 45] with only three including mostly males [36, 37, 43]. Five studies recruited participants through the Jamaica 1986 Birth Cohort Study [33, 37–40], one through the Haiti Cardiovascular Disease Cohort Study [43], and one through the Boricua Youth Study – Health Assessment [45]. Two studies recruited participants through university admission [36, 42] and one study through emergency room attendance [44]. The remaining study recruited participants through a short-term financial assistance initiative offered to families exposed to economic hardship [41]. The majority of studies included objective measurements of NCDs and associated risk factors (*n* = 9) [33, 36–40, 42, 43, 45] with one study including self-reported measurement of NCD prevalence [41] and one study including self-reporting of NCD symptoms [44]. Three studies also included self-reported measures of lifestyle behaviours [33, 42, 45] and one study included objective measurement of sodium intake through urinary excretion [43].

### NCD prevalence

Four studies reported the prevalence of any NCDs [36, 40–42], the most common NCD reported was T2DM with the prevalence ranging from 0.0 to 2.9% [36, 41, 42]. One study reported the prevalence of chronic kidney disease (CKD) as 8.3% [40] and one study reported the prevalence of cardiac disease as 1.0% [41] (Table 2).

### The prevalence of NCD risk factors

Nine studies reported the prevalence of physical risk factors associated with the development of NCDs [33, 36–40, 42, 43, 45].

#### Hypertension

The most commonly reported risk factor was the prevalence of hypertension which ranged from 1.5 to 20.9% [33, 36, 38, 41, 45]. One study indicated that the prevalence of stage 1 hypertension was 10.7% [38] and the prevalence of stage 2 hypertension was 1.3% [38].

#### Anthropometric markers

The prevalence of adverse body composition outcomes were frequently reported with the prevalence of overweight/obesity ranging from 18.8 to 24.9% [36, 38, 45]. One study reported the prevalence of normal weight obesity (NWO) as 20.0% [42] and one study reported the prevalence of central obesity as 15.6% [38].

#### Metabolic markers

Some studies also reported the prevalence of low HDL cholesterol which ranged from 15.9% to 47.0% [33, 36, 45] and one study reported the prevalence of hypercholesterolemia as 0.8% [36]. The prevalence of metabolic syndrome was assessed in two studies with the prevalence ranging from 1.2 to 8.0% [33, 36], and one study demonstrated that 54.0% of participants had at least one component of metabolic syndrome [33]. The remaining risk factors reported were the prevalence of high-sensitivity C-reactive protein concentrations ([hsCRP]) (15.0%) [39], the prevalence of hypertriglyceridemia (30.6%) [36], and the prevalence of hyperlipidaemia (0.4%) [42].

#### Behavioural and lifestyle risk factors

Four studies included data relating to behavioural and lifestyle risk factors of NCDs [33, 42, 43, 45]. One study reported the prevalence of low levels of physical activity (no activity) to be 34.0% and the prevalence of moderate levels of physical activity (≤ 3.5 h per week) to be 42.3% [33]. Additionally, a study reported that 4.2% of participants smoked tobacco, 48.7% consume less than seven alcoholic drinks per week, 2.1% consume one to two alcoholic drinks per day, and 0.4% consume more than five alcoholic drinks per day [42]. Furthermore, one study reported that mean sodium intake was highest in young adults (aged 18–29 years) in comparison to any other age group (5.4 g/day) [43] (Table 2).

#### Gender

Five studies assessed gender differences between the prevalence of physical risk factors with a consensus that females experience a higher prevalence of adverse cardiometabolic risk factors compared to males [36]. Specifically, females had a higher risk of [hsCRP] compared to males (20 vs. 9%) [39]. Females also had a higher prevalence of central obesity compared to males (24.4% vs. 5.1%) [38, 45], were more likely to present with ≥ 1 (68.0%) and ≥ 2 (21.3%) components of metabolic syndrome compared to males (39% and 9% respectively) [33], and had a higher prevalence of low HDL cholesterol compared to males (24.7% vs. 6.1%) [45]. In contrast, males were more likely to present with hypertension compared to females (29.8% vs. 13.4%) [38, 45].

One study reported differences in behavioural and lifestyle risk factors between genders indicating that a higher proportion of females engage in low levels of physical activity compared to males (47.1% vs. 18.0%). Additionally, one study reported differences between geographical locations with participants in New York having greater physical activity (mean scores: 59 vs. 57), lower nicotine exposure scores (mean scores: 70 vs. 86) and lower sleep scores (mean scores: 65 vs. 77) compared to participants in Puerto Rico based on the Life’s Essential 8 score [45].

### Associations

A number of studies assessed the associations between lifestyle factors and cardiometabolic and NCD risk factors [38, 39, 42, 43]. One study indicated that increasing sodium intake from the lowest quartile of consumption (< 3.8 g/day) to the highest quartile (> 5.8 g/day) was associated with an 8.71 mmHg increase in systolic blood pressure (BP) [43]. Additionally, a study demonstrated that physical activity was negatively associated with hypertension [38]. Another study demonstrated that increased [hsCRP] were positively related to waist circumference (WC) and having an increased number of metabolic syndrome components [39]. Furthermore, a study indicated that participants with NWO were significantly more likely to have elevated systolic BP and higher total cholesterol/HDL cholesterol ratio (TC/HDL), and as such are at a higher cardio-metabolic risk compared to those with normal body fat levels [42] (Table 2).

Few studies also assessed the associations between demographic and cardiometabolic NCD risk factors [37–40]. One study demonstrated that a one standard deviation increase (approximately 550 g) in birthweight was associated with a 2.2% increase in estimator glomerular filtration rate (eGFR) [40]. Another study indicated that gestational age and birth weight were related to increased nocturnal salivary cortisol concentrations ([CORT]) [37]. A different study indicated that lower parental education was associated with increased [hsCRP] [39]. Furthermore, a study showed that the prevalence of hypertension was positively related to fewer household possessions in females [38] (Table 2).

Several studies investigated differences in associations between males and females [38, 39, 42]. One study showed that the prevalence of hypertension was positively associated with obesity and high blood glucose ([Glu]) among males, and with high triglycerides and high homeostatic model for insulin resistance among females (HOMA-IR) [38]. The same study also showed that alcohol consumption was negatively related with hypertension in females only [38]. Furthermore, a study showed that increased [hsCRP] were positively related to body mass index (BMI) category, high WC, high triglyceride concentrations ([TG]), low HDL cholesterol, among females, but only for high WC among males [39] (Table 2).

Finally, one study assessed the association between environmental factors and NCD symptomology with an increased number of asthma-related emergency room visits occurring during months with increased rainfall, when Saharan dust is of a higher concentration [44] (Table 2).

**Table 2:** Characteristics of studies included in the review (*n* = 11)

Reference	Country of study	Study design	Sample size	Sample description	Outcome measures	Key findings
Bennett et al., 2014 [39]	Jamaica	Cross sectional study	746	Females (54%) Aged 18–20 Recruited from the Jamaica 1986 Birth Cohort Study	[hsCRP] BMI BP WC [Glu] [TC] [LDL] [HDL] [TG]	15% high-risk [hsCRP] (> 3 mg/L), with females showing a higher prevalence than males (20% vs. 9%). In females, high [hsCRP] was associated with higher BMI, larger WC, elevated [TG], lower [HDL], and lower parental education. For males, it was associated with high WC and lower parental education. High WC significantly increased the risk of elevated [hsCRP] (OR 7.8, 95% CI 4.8–12.9). High [hsCRP] was also associated with a higher number of metabolic syndrome components. Individuals with one component had twice the odds of high [hsCRP] compared to those with none (OR 2.2, 95% CI 1.3–3.8), while those with three components had a 14 times higher risk compared to those with none(OR 13.5, 95% CI 2.4–76.0).
Rocke et al., 2018 [40]	Jamaica	Longitudinal cohort study	744	Females (54%) Aged 18–20 Recruited from the Jamaica 1986 Birth Cohort Study	Prevalence of CKD BMI BP [Glu] [HDL] [TG] Creatinine concentrations [Cr] Urinary albumin concentrations [Alb] eGFR Birth weight	The prevalence of CKD was 8.3%. eGFR was associated with birthweight. A one standard deviation increase (approximately 550 g) in birthweight was associated with a 2.2% increase in eGFR. Participants with a low birthweight were found to have a 5.1% reduction in eGFR.
Clermont et al., 2023 [43]	Haiti	Population-based cohort study	1,240	Females (48%) Aged 18–93 427 participants aged 18–29 Recruited from the Haiti Cardiovascular Disease Cohort Study	Dietary sodium intake via 24-hour urinary sodium excretion	Mean sodium intake was highest in 18–29 year olds (5.4 g/day) In 18–29 year olds, increasing salt intake from the lowest quartile of consumption (< 3.8 g/day) to the highest quartile (> 5.8 g/day) was associated with an 8.71 mmHg higher systolic BP
Ferguson et al., 2018 [38]	Jamaica	Cross sectional study	898	Females (54%) Aged 18–20 Recruited from the Jamaica 1986 Birth Cohort Study	BMI BP [Glu] [TC] [LDL] [HDL] [TG] [hsCRP] [Insulin] HOMA-IR	20.9% had elevated BP (BP ≥ 120/80mmHg) 10.7% stage 1 hypertension (BP 130–139/80-89mmHg) 1.3% stage 2 hypertension (BP ≥ 140/90mmHg) 24.9% overweight/obese (BMI ≥ 25.0 kg/m2) 15.6% central obesity The prevalence of hypertension was higher among males (29.8%) than females (13.4%) The prevalence of central obesity was higher among females (24.4%) than males (5.1%) The prevalence of hypertension was positively associated with obesity and high [Glu] among males, and with high triglycerides, high HOMA-IR and fewer household possessions among females Higher levels of HOMA-IR was also associated with hyptertension among males, but this did not achieve statistical significance. Physical activity was inversely associated with hypertension in both males and females, while alcohol consumption was inversely associated with hypertension in females only
Ferguson et al., 2010 [33]	Jamaica	Cross sectional study	839	Females (55%) Aged 18–20 Recruited from the Jamaica 1986 Birth Cohort Study	BMI Waist circumference Hip circumference BP [Glu] [TC] [LDL] [HDL] [TG] Self-reported physical activity Prevalence of metabolic syndrome	1.2% metabolic syndrome 54.0% one component of metabolic syndrome Low [HDL] was the most common component present (47.0%) Females were more likely to present with ≥ 1 (68%) and ≥ 2 (21.3%) components of metabolic syndrome compared to males (39% and 9.0% respectively) 34.0% reported low levels of physical activity with a higher prevalence in females (47.1%) than males (18.0%) 42.3% reported moderate levels of physical activity (≤ 3.5 h per week) with a higher prevalence in males (45.2%) than females (39.7%)
Ramsaran et al., 2017 [42]	Trinidad and Tobago	Cross sectional study	236	Females (68%) Aged 18–28 First year university students	BMI Waist circumference Hip circumference Body fat percentage BP [Glu] [TC] [LDL] [HDL] [TG] [HbA1C] [hsCRP] Self-reported lifestyle behaviours (i.e., tobacco and alcohol use) Prevalence of NWO	1.3% diabetes mellitus 0.4% hyperlipidaemia 20.0% NWO 4.2% smoke tobacco 48.7% consume < seven alcoholic drinks per week 2.1% consume one to two alcoholic drinks per day 0.4% consume more than five alcoholic drinks per day Young adults with NWO were significantly more likely to have elevated systolic BP and higher TC/HDL ratio, and as such are at a higher cardio-metabolic risk compared to those with normal body fat levels.
Chadee et al., 2013 [41]	Trinidad and Tobago	Retrospective database study	14,793	Females (86%) 1,835 participants aged 17–30 Recruited through a short-term financial assistance programme	Prevalence of self-reported diabetes Prevalence of self-reported hypertension Prevalence of self-reported cardiac disease	2.9% T2DM 7.5% hypertension 1.0% cardiac disease Females had a greater prevalence than males for all diseases
Thompson et al., 2015 [37]	Jamaica	Observational cohort study	60	Females (45%) Aged 18–20 Recruited from the Jamaica 1986 Birth Cohort Study	BMI Waist circumference Body composition (measured using DXA) Serum [andipotencin] Serum [IGF-1] Serum myostatin concentrations [MSTN] Salivary [CORT] [Glu] [Insulin]	Gestational age and birth weight were positively associated with nocturnal salivary cortisol in young adulthood Higher levels of salivary cortisol was associated with reduced glucose effectiveness
Akpinar-Elci et al., 2015 [44]	Grenada	Retrospective cohort study	4411	Females (53%) Mean age 20 ± 2 Asthma emergency room attendees	Daily and monthly mean of asthma-related visits to emergency room Sharan dust volumes	Increase in number of asthma-related emergency room visits during rainy months when Saharan dust is of a higher concentration
Martinez at al., 2017 [36]	Cuba	Analytical cross-sectional study	242	Females (40%) Aged 18–19 University students	BMI Waist circumference Hip circumference Body fat percentage BP [Glu] [HDL] [TG] Prevalence of T2DM and pre-diabetes Prevalence of hypercholesterolemia Prevalence of hypertriglyceridemia Prevalence of hypertension Prevalence of abdominal obesity Prevalence of overweight/obesity Prevalence of metabolic syndrome	22.3% had abdominal obesity 0.0% had T2DM or pre-diabetes 19.8% had overweight or obesity 14.0% had hypertension 30.6% had hypertriglyceridemia 23.1% had low [HDL] 0.8% had hypercholesterolemia 8.3% had metabolic syndrome The presence of adverse cardiometabolic risk factors was more likely in females compared to males.
Suglia et al., 2024 [45]	USA and Puerto Rico	Longitudinal study	759	Females (51%) Mean age 23 ± 0 413 participants from Puerto Rico Recruited from the Boricua Youth Study – Health Assessment	BMI BP [Glu] [HDL] [HbA1c] Self-reported lifestyle behaviours (e.g., dietary and physical activity behaviours)	1.5% hypertension 19.9% high [HbA1C] (≥ 5.7mmol/mol) 18.8% obese 15.9% low [HDL] Higher prevalence of low [HDL] cholesterol in Puerto Rico (15.9%) than New York (7.2%) Lower prevalence of obesity in Puerto Rico (18.8%) than New York (34.7%) Lower prevalence of hypertension in Puerto Rico (1.5%) than New York (7.9%) Men had a higher prevalence of hypertension compared to women (3.0% vs. 0.0%) Women had a higher prevalence of obesity (21.1% vs. 16.4%) and low [HDL] compared to men (24.7% vs. 6.1%) Nicotine exposure was higher in Puerto Rico (mean score: 86) than New York (mean score: 70) Physical activity was also lower in Puerto Rico (mean score: 57) than New York (mean score: 59) Sleep scores were higher in Puerto Rico (mean score: 77) than New York (mean score: 65) Women had better diet and nicotine exposure scores compared to men Women had lower BP, more favourable lipid profiles and better physical activity scores compared to men

## Discussion

The aim of the current review was to explore the existing evidence base surrounding NCDs in young adults in the Caribbean. Additionally, the comprehensive search strategy employed in the current scoping identified only eleven studies that collected data surrounding NCD risk and prevalence in this population. Of the twenty-six Caribbean islands, only six were represented among the studies included with the majority (*n* = 5) set in Jamaica. Participants in the studies were aged 17–30 and were recruited through three large, existing cohort studies, a government-led financial initiative, and through universities. Only four studies assessed the prevalence of NCDs with nine reporting the prevalence of physical risk factors, and four reporting behavioural risk factors.

### NCD prevalence

This review highlights the prevalence of some NCDs in young adults across the Caribbean. The prevalence of T2DM was the most commonly reported NCD (*n* = 3 studies) and ranged between 0.0 and 2.9% [36, 41, 42]. One study also reported the prevalence of CKD as 8.3% [40] and another study indicated the prevalence of cardiac disease was 1.0% [41]. The prevalence of NCDs has increased substantially across the globe in recent times; however, the Caribbean islands have experienced a disproportionately steep increase in those developing these conditions [4, 46]. Specifically, the prevalence of T2DM in all age groups across the Caribbean is stated to be 15.0% [46] in comparison to the global prevalence of 6.3% [47]. Additionally, the prevalence of CKD in the Caribbean is 11.9% compared to 10% across the globe [48], and the incidence of cardiovascular disease is higher in the Caribbean compared to other countries around the world (123 per 100,000 in Barbados vs. 63.1 per 100,000 in Japan) [49]. This may be due to genetic factors that pre-dispose those from Black and Caribbean ethnic backgrounds to increased risk of NCDs such as T2DM [50], but it is also likely that adverse lifestyle changes such as physical inactivity, increased sedentary behaviour and poorer dietary choices (e.g., increased intake of fast foods, sugar-sweetened beverages and processed meat) and rapid urbanisation play a role in the aetiology of the disease [51]. As such, it is promising that the prevalence of young adults presenting with NCDs included in the current review is lower than Caribbean and global general populations [46–49]. While this may be interpreted as a positive finding, NCDs develop as a results of chronic adverse health-related behaviours meaning they are typically initiated when young and are exacerbated later into adulthood [52]. This extended delay in the onset of such disorders may mean that assessing NCD prevalence alone may not provide sufficient detail surrounding health status in a young adult population. Instead, evaluating physical factors that act as precursors to the development of NCDs may be more informative. Nine studies in the current review assessed the prevalence of physical risk factors for NCDs [33, 36–40, 42, 43, 45].

### Hypertension

The prevalence of hypertension was examined in five studies and ranged between 1.5 and 20.9% [33, 36, 38, 41, 45] with one study indicating the prevalence of stage one hypertension as 10.7% and stage two hypertension as 1.3% [38]. The large range in prevalence data may be due to the methods employed to define high BP. For instance, the study with the lowest prevalence (2%) defined hypertension as systolic BP ≥ 130 mmHg, or diastolic BP ≥ 85 mmHg, or both [45] whereas the study with the highest prevalence (20.9%) used a cut-off of ≥ 120/80mmHg to define elevated BP [38]. Furthermore, another study classified hypertension as 140/90mmHg [33] and one study asked participants to self-report hypertension without providing stipulations as to how hypertension was defined [41]. Given the large variation in methods of classifying hypertension, it is difficult to synthesise current literature on this topic. Nonetheless, the prevalence of hypertension is increasing across the globe in all age groups [53–55] and it is therefore likely that similar trends are occurring in young adults across the Caribbean [56].

### Overweight/obesity

The prevalence of adverse body composition outcomes also reported by four studies with the prevalence of overweight/obesity ranging from 18.8 to 24.9% [36, 38, 42, 45]. Again however, studies included utilised a range of methodologies and guidelines to define overweight/obesity providing challenges to literature synthesis. Two studies used standardised BMI classifications (overweight: 25.0–29.9 kg/m^2^, obese: ≥30.0 kg/m^2^) [38, 45], one study used IDF guidelines (BMI ≥ 30.0 kg/m^2^ & WC ≥ 102 cm in men and ≥ 88 cm in women) [36] and another study examined NWO using a BMI between 18.5 and 24.9 kg/m^2^ in combination with body fat ≥23.1% in men or ≥33.3% in women [42]. Whilst it is difficult to compare these findings, the range of prevalence data is similar to data from 2014 to 2016 in Caribbean children and adolescents (aged 6–20 years) that indicates the prevalence of overweight as between 10.6 and 21.2% and obesity as between 7.1 and 25.4% [57]. Promisingly, these figures are lower than those proposed in young adults from westernised countries such as the UK where the prevalence of overweight/obesity in young adults is suggested to be 37.0% [58]. Nonetheless, the small number of studies included in the current review and the range of methodologies used make comparisons difficult. Additionally, the Caribbean islands are suggested to have experienced some the highest increases in overweight and obesity in recent times [59] and further data are required to understand whether young adults in the region are following this trend.

### Other physical NCD risk factors

The prevalence of metabolic syndrome was reported in two studies and ranged from 1.2 to 8.0% [33, 36]. One study indicated that the prevalence of young adults with at least one component of metabolic syndrome was 54.0% [33]. In line with this, three studies reported the prevalence of low HDL cholesterol (15.9–47.0%) and one study reported the prevalence of hypertriglyceridemia (30.6%) [36]. Other physical risk factors reported in the current review were the prevalence of increased [hsCRP] (15.0%) [39], the prevalence of hypercholesterolemia (0.8%) [36], and the prevalence of hyperlipidaemia (0.4%) [42]. Taken together, these findings suggest that less than 10% of young adults in the Caribbean are presenting with metabolic syndrome, data that is in agreement with a recent global review (metabolic syndrome prevalence: 4.8-7.0%). However, in contrast, these data indicate that over half have at least one component of metabolic syndrome in comparison to a third of young adults from across the globe [35]. Although overall metabolic syndrome prevalence is seemingly low, the high prevalence of one metabolic syndrome component is concerning given that this often leads to the development of further components, and subsequently complete metabolic syndrome [35, 60]. Interestingly, the most common component of metabolic syndrome reported in the current review was low HDL cholesterol which is in agreement with previous literature (low HDL cholesterol prevalence: 26.9–41.2%) [35]. Low levels of HDL cholesterol primarily result from an increase in triglyceride production, which diminishes the cholesterol content within the lipoprotein core [61]. Although the mechanisms are currently unknown, it is speculated that the development of low HDL cholesterol may initiate a series of pathophysiological processes that increase the likelihood of developing further components of metabolic syndrome in young adults [35]. Therefore, further research is required to understand why low HDL cholesterol is the most frequent feature of metabolic syndrome in young adults, and why young adults from the Caribbean are seemingly at greater risk of having at least one component of metabolic syndrome.

### Behavioural risk factors

The development of NCDs and related risk factors is largely driven by chronic adverse behaviours that become habitual throughout the lifespan [1, 2]. However, only four of the studies identified in this review assessed behavioural outcomes in addition to NCD risk [33, 42, 43, 45]. Findings indicated that a third of Caribbean young adults (34.0%) do not engage in any physical activity and 4.2% smoke tobacco [33, 42]. Additionally, a study found that young adults consume more sodium (5.4 g/day) than any other age group [43] while a different study stated that 48.7% consume alcohol during the week (less than seven drinks containing alcohol) [42]. Although limited, these data suggest that substantial proportions of young adults across the region engage in adverse health-related behaviours. It is likely that these behaviours are driven by a multitude of social, cultural, environmental, and economic factors stemming from increasing urbanisation across the Caribbean region [10]. Nonetheless, these findings are particularly concerning given that young adulthood is a critical period for developing health-related habits and behaviours that can have a profound influence on outcomes of health and behaviours throughout the lifespan [22–28]. This is evidenced further through associations identified in the current review suggesting that poorer behaviours (e.g., less physical activity) were related to worse health markers (e.g., elevated BP) [38, 39, 42, 43].

Implementing initiatives aimed at improving these behaviours in this population may therefore aid in reducing the current and future burden of NCDs across the Caribbean. In 2007, the Heads of Government of the Caribbean Community (CARICOM) affirmed their commitment to improving NCD prevention and control through the Declaration of Port-of-Spain [62]. While progress has been made, a recent evaluation highlights ongoing challenges in achieving targets related to physical activity and dietary habits, as reported in WHO global monitoring documents [63]. In response, individual CARICOM nations have introduced nationwide interventions, such as the “Jamaica Moves” campaign and taxes on sugar-sweetened beverages (SSBs), designed to promote regular physical activity and healthier eating habits. Preliminary evidence suggests some success, including reductions in the purchase and consumption of SSBs [64]. However, the broad scope of these initiatives makes it difficult to evaluate their effectiveness among specific groups, such as young people. Additionally, the current scoping review highlights gender disparities in NCD risk with females generally experiencing a greater prevalence of physical NCD risk factors (other than hypertension) compared to males [33, 36, 38, 45, 50]. This is suggested to be due to an increase in obesity in young women but it is possible that underlying conditions (e.g., polycystic ovary syndrome) leading to dysregulation of sex hormones (i.e., reducing oestrogen and increasing testosterone) that play a role in physiological processes and alter risk factors for disease such as reduced insulin resistance are also contributing to this increase [65]. Given these differences, a “one-size-fits-all” approach to interventions is unlikely to achieve optimal engagement or effectiveness. Therefore, the design of new initiatives should account for gender-specific factors that incorporate culturally tailored approaches (e.g., creating safe and supportive environments for females to engage with physical activity) to enhance their impact on reducing NCD risk.

### Implications

This scoping review highlights the severe lack of data surrounding NCD risk and prevalence in young adults across the Caribbean. This may be due to a lack of adequate surveillance systems to monitor health-based statistics across the region. Historically, the Caribbean islands have faced environmental challenges to accessing locally stored and paper-based data due to environmental factors (e.g., climate change) that lead to damage or complete loss [66]. However, recent efforts have improved access to digitised data [67]. Consequently, population-based datasets are becoming more accessible; however, they primarily examine cancer risk and prevalence, with limited attention given to young adults [68]. Moreover, while novel frameworks to assess communicable diseases are currently being implemented [69], they lack comprehensive data on behavioural factors that may contribute to the development of NCDs, particularly among young adults. Generating more information on this topic is vital considering that the Caribbean islands are currently experiencing a disproportionately high level of burden related to NCDs and this trend is expected to continue in the future [8, 9, 70].

Limitations of the current scoping review include the scope of data sources being limited to major databases (e.g., PubMed) and publications available primarily in English. This may have excluded studies written in other languages (e.g., Dutch & French) that are commonly used in some Caribbean islands which could lead to publication bias and restrict the external validity of the findings. Additionally, the concepts of NCD risk and prevalence are complex and incorporate a plethora of terminology. As such, it is possible that some search terms may have been omitted during the research process. However, the substantial number of articles retrieved in the initial search suggests that the search strings used were sufficiently broad to capture the majority of relevant literature in the field. Furthermore, almost half of the studies included in this review (5 of 11) were conducted in Jamaica, with only 6 of the 26 Caribbean islands represented overall. This limited geographical distribution may reduce the external validity of the findings for the wider Caribbean region. Nonetheless, the literature search was comprehensive and conducted in accordance with the appropriate processes. While this limitation may constrain generalisability of the findings, it also underscores the paucity of research across the Caribbean and highlights the need for more concerted efforts to generate evidence from a broader range of islands within the region. Finally, a key limitation of this review is the considerable methodological heterogeneity across the included studies. These inconsistencies limit the comparability of prevalence estimates and introduce potential bias in the interpretation of findings. Future research would benefit from the adoption of standardised definitions and measurement approaches, which would enhance comparability across studies and strengthen the evidence base for regional risk factor profiles. However, given the small number of studies included in the review and the considerable social, cultural, economic, and environmental differences across geographical settings, a degree of heterogeneity was inevitable.

## Conclusion

This scoping review highlights the current landscape related to NCD risk and prevalence in young adults across the Caribbean. Although the prevalence of NCDs and physical risk factors appear to be low, there is a severe paucity of data available in the area, resulting in a lack of representation from the majority of Caribbean islands and inconclusive findings. Enhancing existing surveillance systems to incorporate data surrounding NCD risk and prevalence, specifically in young adults, will aid in assessing the efficacy of current health-based interventions and support the development of novel initiatives to ultimately reduce the current and future burden of NCDs within the Caribbean islands.

## Supplementary Information

Supplementary Material 1.

Supplementary Material 2.

## Data Availability

Inquiries related to data availability should be addressed to the corresponding authors of each publication included in the current review.

## Abbreviations

NCD: Non-communicable disease
CKD: Chronic kidney disease
T2DM: Type 2 diabetes mellitus
PRISMA-ScR: Preferred Reporting Items for Systematic Reviews and Meta-Analyses Scoping Reviews
BMI: Body mass index
BP: Blood pressure
NWO: Normal weight obesity
WC: Waist circumference
hsCRP: High-sensitivity C-reactive protein concentration
CORT: Cortisol concentration
HOMA-IR: Homeostatic model for insulin resistance
Glu: Glucose concentration
TC: Total cholesterol concentration
LDL: Low-density lipoprotein concentration
HDL: High-density lipoprotein concentration
TG: Triglyceride concentration
MSTN: Myostatin concentration
