# Supplementary material for: The prevalence of non-communicable diseases and related risk factors in young adults in the Caribbean islands: a scoping review
Source: BMC Public Health. 2026 Mar 28;26:1490. doi: 10.1186/s12889-026-27072-2 (PMC13151328; doi:10.1186/s12889-026-27072-2)
Supplement: Supplementary file 2 — Supplementary Material 2. [file 12889_2026_27072_MOESM2_ESM.docx]

**Supplementary data 2. Search string for each database**

**PubMed**

**(("young adults"[Title/Abstract] OR "adolescents"[Title/Abstract] OR "young people"[Title/Abstract] OR "young person"[Title/Abstract]) AND ("non communicable diseases"[Title/Abstract] OR "NCDs"[Title/Abstract] OR "noncommunicable disease"[Title/Abstract] OR "noninfectious diseases"[Title/Abstract] OR "noninfectious disease"[Title/Abstract] OR "non infectious diseases"[Title/Abstract] OR "non infectious disease"[Title/Abstract] OR "non infectious diseases"[Title/Abstract] OR "non communicable diseases"[Title/Abstract] OR "disease non communicable"[Title/Abstract] OR "non communicable disease"[Title/Abstract] OR "non communicable diseases"[Title/Abstract] OR "non communicable chronic diseases"[Title/Abstract] OR "chronic disease non communicable"[Title/Abstract] OR "non communicable chronic disease"[Title/Abstract] OR "non communicable chronic diseases"[Title/Abstract] OR "non communicable illness"[Title/Abstract] OR "non communicable disorders"[Title/Abstract] OR "Asthma"[Title/Abstract] OR "heart disease"[Title/Abstract] OR "Stroke"[Title/Abstract] OR "heart failure"[Title/Abstract] OR "Hypertension"[Title/Abstract] OR "Diabetes"[Title/Abstract] OR "chronic kidney disease"[Title/Abstract] OR "mental health disorders"[Title/Abstract]) AND ("Caribbean"[Title/Abstract] OR "caribbean region"[Title/Abstract] OR "caribbean islands"[Title/Abstract] OR "Anguilla"[Title/Abstract] OR "Antigua"[Title/Abstract] OR "Barbuda"[Title/Abstract] OR "Aruba"[Title/Abstract] OR "the bahamas"[Title/Abstract] OR "Barbados"[Title/Abstract] OR "british virgin islands"[Title/Abstract] OR "cayman islands"[Title/Abstract] OR "Cuba"[Title/Abstract] OR "Dominica"[Title/Abstract] OR "dominican republic"[Title/Abstract] OR "Grenada"[Title/Abstract] OR "Guadeloupe"[Title/Abstract] OR "Haiti"[Title/Abstract] OR "Jamaica"[Title/Abstract] OR "Martinique"[Title/Abstract] OR "netherlands antilles"[Title/Abstract] OR "puerto rico"[Title/Abstract] OR "st barts"[Title/Abstract] OR "st kitts"[Title/Abstract] OR "Nevis"[Title/Abstract] OR "st lucia"[Title/Abstract] OR "st martin"[Title/Abstract] OR "st vincent"[Title/Abstract] OR "Trinidad"[Title/Abstract] OR "Tobago"[Title/Abstract] OR "Turks"[Title/Abstract] OR "Caicos"[Title/Abstract])) NOT ("Children"[Title/Abstract] OR "middle-aged"[Title/Abstract] OR "older adults"[Title/Abstract] OR "kids"[Title/Abstract] OR "elderly"[Title/Abstract] OR "cancer"[Title/Abstract])**

Number of articles: 52

**Web of Science**

TS=("young adults" OR "adolescents" OR "young people" OR "young person")

AND TS=("non communicable diseases" OR "NCDs" OR "noncommunicable disease" OR "noninfectious diseases" OR "noninfectious disease" OR "non infectious diseases" OR "non infectious disease" OR "non infectious diseases" OR "non communicable diseases" OR "disease non communicable" OR "non communicable disease" OR "non communicable diseases" OR "non communicable chronic diseases" OR "chronic disease non communicable" OR "non communicable chronic disease" OR "non communicable chronic diseases" OR "non communicable illness" OR "non communicable disorders" OR "Asthma" OR "heart disease" OR "Stroke" OR "heart failure" OR "Hypertension" OR "Diabetes" OR "chronic kidney disease" OR "mental health disorders")

AND TS=("Caribbean" OR "caribbean region" OR "caribbean islands" OR "Anguilla" OR "Antigua" OR "Barbuda" OR "Aruba" OR "the bahamas" OR "Barbados" OR "british virgin islands" OR "cayman islands" OR "Cuba" OR "Dominica" OR "dominican republic" OR "Grenada" OR "Guadeloupe" OR "Haiti" OR "Jamaica" OR "Martinique" OR "netherlands antilles" OR "puerto rico" OR "st barts" OR "st kitts" OR "Nevis" OR "st lucia" OR "st martin" OR "st vincent" OR "Trinidad" OR "Tobago" OR "Turks" OR "Caicos")

NOT TS=("Children" OR "middle-aged" OR "older adults" OR "kids" OR "elderly" OR "cancer")

Number of articles: 53

**Scopus**

**(TITLE-ABS-KEY ( "young adults" OR "adolescents" OR "young people" OR "young person") AND TITLE-ABS-KEY ( "non communicable diseases" OR "NCDs" OR "noncommunicable disease" OR "noninfectious diseases" OR "noninfectious disease" OR "non infectious diseases" OR "non infectious disease" OR "non infectious diseases" OR "non communicable diseases" OR "disease non communicable" OR "non communicable disease" OR "non communicable diseases" OR "non communicable chronic diseases" OR "chronic disease non communicable" OR "non communicable chronic disease" OR "non communicable chronic diseases" OR "non communicable illness" OR "non communicable disorders" OR "Asthma" OR "heart disease" OR "Stroke" OR "heart failure" OR "Hypertension" OR "Diabetes" OR "chronic kidney disease" OR "mental health disorders" ) AND TITLE-ABS-KEY ( "Caribbean" OR "caribbean region" OR "caribbean islands" OR "Anguilla" OR "Antigua" OR "Barbuda" OR "Aruba" OR "the bahamas" OR "Barbados" OR "british virgin islands" OR "cayman islands" OR "Cuba" OR "Dominica" OR "dominican republic" OR "Grenada" OR "Guadeloupe" OR "Haiti" OR "Jamaica" OR "Martinique" OR "netherlands antilles" OR "puerto rico" OR "st barts" OR "st kitts" OR "Nevis" OR "st lucia" OR "st martin" OR "st vincent" OR "Trinidad" OR "Tobago" OR "Turks" OR "Caicos" ) AND NOT TITLE-ABS-KEY ( "Children" OR "middle-aged" OR "older adults" OR "kids" OR "elderly" OR "cancer"))**

**Number of articles: 308**
